# Associations of Pregnancy Physical Activity with Maternal Cardiometabolic Health, Neonatal Delivery Outcomes and Body Composition in a Biethnic Cohort of 7305 Mother–Child Pairs: The Born in Bradford Study

**DOI:** 10.1007/s40279-019-01193-8

**Published:** 2019-09-26

**Authors:** Paul J. Collings, Diane Farrar, Joanna Gibson, Jane West, Sally E. Barber, John Wright

**Affiliations:** 1 Bradford Institute for Health Research, Bradford Teaching Hospitals NHS Foundation Trust, Bradford, UK; 2 Department of Health Sciences, University of York, York, UK

## Abstract

**Objective:**

Physical activity is advocated for a range of benefits to the uncomplicated pregnancy. We investigated associations of mid-pregnancy physical activity with maternal and neonatal health in white British and Pakistani-origin women from a deprived urban setting.

**Methods:**

The study was performed in 6921 pregnant women (53% Pakistani-origin) who contributed data for 7305 singleton births. At 26–28 weeks gestation, women were grouped into four activity levels (inactive/somewhat active/moderately active/active) based on their self-reported physical activity. Linear regression with robust standard errors was used to calculate adjusted mean differences in health markers between the four groups of physical activity (reference group: inactive).

**Results:**

Three-quarters (74%) of Pakistani-origin women and 39% of white British women were inactive. Trend-tests revealed that more active white British women tended to be less adipose, had lower fasting and postload glucose levels, lower triglyceride concentrations, and their babies were less adipose (smaller triceps and subscapular skinfolds) than less active white British women. Somewhat active Pakistani-origin women exhibited lower triglyceride concentrations and systolic blood pressure, higher high-density lipoprotein cholesterol levels, and their babies were less adipose (smaller mid-upper arm and abdominal circumferences; lower cord-blood leptin concentration) compared to inactive Pakistani-origin women. No associations were observed for gestational age or birth weight.

**Conclusions:**

Physical activity performed mid-pregnancy was beneficially associated with maternal cardiometabolic health and neonatal adiposity, without influencing gestational age or birth weight. Associations were dose-dependent in white British women, and even a small amount of mid-pregnancy physical activity appeared to benefit some health markers in Pakistani-origin women.

**Electronic supplementary material:**

The online version of this article (10.1007/s40279-019-01193-8) contains supplementary material, which is available to authorized users.

## Key Points

**Table:** 

1. Few studies have investigated associations of pregnancy physical activity with maternal and neonatal health markers in minority ethnic and economically disadvantaged groups of women, who are higher risk for pregnancy complications.
2. We found that mid-pregnancy physical activity was beneficially associated with maternal cardiometabolic health markers and neonatal adiposity in white British and Pakistani-origin women living in a deprived urban setting, without influencing gestational age or birth weight.
3. Associations were dose-dependent in white British women. In Pakistani-origin women (three-quarters of whom were inactive) even a small volume of physical activity in mid-pregnancy conferred some health benefit. This could be influential in helping to reduce health inequalities.

## Introduction

Regular physical activity is advocated for a range of benefits to the uncomplicated pregnancy. This includes reduced risk of complications during birth [1], excess gestational weight gain, gestational diabetes, and hypertensive disorders [2, 3], factors that can have long-term adverse effects for mother and child [4–6]. Mechanisms of action have been minimally investigated, but are expected to include favourable changes in adiposity, insulin sensitivity, blood pressure, blood lipid and lipoprotein concentrations [7, 8]. Children born to active mothers may also benefit from reduced risk of prematurity [9, 10], reduced risk of being small- or large- for gestational age [10, 11], and a lower fat mass at birth [12].

Broadly in line with recommendations for the general adult population, national guidelines state that over the course of a week pregnant women should perform 150 min of moderate intensity physical activity that is both safe and comfortable [13–15]. Few meet this recommendation, with recent rates of compliance ranging from only 3 to 35% [16–20]. As in the general population, evidence suggests that non-white women (of black, Hispanic and Asian ethnicity living in America and Europe) self-report lower levels of physical activity in pregnancy [21]. This has been replicated using objective measurements [20, 22]. In a multi-ethnic Norwegian sample measured at 28 weeks gestation, South Asian women (mostly of Pakistani origin) were nearly 3 times more likely be physically inactive than Western European women [20]. Although causes are likely multiple, it is possible that low levels of pregnancy physical activity may partly explain why Pakistani-origin women are at increased risk of developing gestational [23] and type 2 diabetes and cardiovascular disease [24, 25], and why children of Pakistani origin are born with greater adiposity for equivalent birth size compared to white Europeans [26, 27].

Studies evaluating the effects of pregnancy physical activity on health have almost exclusively been performed in samples of well-educated white American or European women [3, 21]. Information regarding health associations in specific high-risk populations, such as minority ethnic and economically disadvantaged groups of women is lacking [2]. Filling these gaps is important to aid understanding of health inequalities. This is particularly true for Pakistani-origin women living in the UK, who are susceptible to increased health risks from a combination of both ethnicity and deprivation (in England, in 2011, over 50% of Pakistani-origin people lived in the most deprived 20% of areas [28]). To determine if ethnicity-specific guidelines and interventions warrant consideration, studies of differential effects of physical activity between white and non-white pregnancies in representative population-based samples are needed. This information could help to reduce health inequalities by informing interventions aimed at preventing adverse pregnancy outcomes and their long-term sequelae in high-risk groups.

The purpose of this study was to investigate associations of mid-pregnancy physical activity with maternal cardiometabolic health markers (adiposity, gestational weight gain, blood glucose and insulin, blood pressure, cholesterol levels), neonatal delivery outcomes (gestational age and birth weight) and neonatal body composition, in a population-based cohort of white British and Pakistani-origin women from an economically deprived city in the UK.

## Methods

### Participants

Born in Bradford (BiB), a prospective birth cohort study, recruited 12,453 women who delivered 13,818 live births from 13,776 pregnancies between March 2007 and December 2010. Women were primarily recruited at 26–28 weeks gestation while they attended an oral glucose tolerance test (OGTT) that is offered to all women booked to deliver at Bradford Royal Infirmary, the only maternity unit serving the city. Bradford is the sixth largest metropolitan borough in England and includes some of the most deprived areas in the country [28]. All women were eligible to take part in the project. The BiB cohort was broadly representative of the obstetric population in Bradford at the time of recruitment [29]. Ethical approval was granted by Bradford Research Ethics Committee (07/H1302/112) and all mothers provided informed written consent. The study was performed in accordance with the ethical standards of the Declaration of Helsinki.

This investigation necessitated maternally reported physical activity which was available for 9621 pregnancies (questionnaires were completed for 11,395 pregnancies, but a first version of the questionnaire that did not cover physical activity was completed for 1571 pregnancies; missing physical activity data for another 203 pregnancies were incidental). All analyses were restricted to the two majority ethnic groups of Pakistani-origin (*n *= 4347) and white British women (*n *= 3795). Women from other South Asian ethnicities, including Indian (*n *= 377) and Bangladeshi (*n *= 204) were excluded due to small numbers, as were women from other white backgrounds and mixed ancestry. Additionally, only women known to be free from pre-existing hypertension and diabetes prior to pregnancy (*n *= 7524) and carrying a singleton pregnancy were included (*n *= 7305). The analytical sample for this study comprised 6,921 pregnant women (52.9% Pakistani-origin) who contributed data for 7305 singleton births.

### Pregnancy Physical Activity

Maternal physical activity performed mid-pregnancy was assessed at recruitment using the General Practice Physical Activity Questionnaire (GPPAQ) which has been validated against accelerometry [30]. Based on the European Prospective Investigation into Cancer (EPIC) physical activity questionnaire, the abbreviated GPPAQ combines information regarding occupational physical activity, physical exercise (such as swimming and aerobics) and cycling performed in the last week into a four-level index (inactive/somewhat active/moderately active/active). We further included walking and perceived usual walking pace in the composite physical activity index (see Supplementary file 1 for the scoring algorithm) [31]. The active category is consistent with achieving the recommended minimum of 150 min of moderate intensity physical activity per week [13–15, 30]. For non-English speaking women the questionnaire was administered in their preferred language. The GPPAQ has previously been implemented in diverse groups following translation to non-English [32].

### Maternal Cardiometabolic Health

At recruitment (26–28 weeks gestation) a 75 g OGTT was carried out according to modified WHO recommendations that were in operation at the time [33]. Fasting blood insulin and glucose concentrations, and 2 h postload glucose levels were determined, and an additional fasting blood sample was taken for research purposes and analysed for blood lipids. Blood pressure was routinely measured by trained obstetricians and midwives according to UK recommendations [34]. Trained researchers measured mid-upper arm circumferences and maternal triceps skinfold thickness was measured using Holtain Tanner/Whitehouse Calipers (Holtain Ltd, Crymych). Late-pregnancy gestational weight gain was calculated as weight measured at recruitment subtracted from weight in the third trimester, which was abstracted from antenatal medical records (third trimester weight was measured on average at 36 weeks gestation; the mean ± standard deviation measurement interval was 10.5 ± 2.4 weeks).

### Neonatal Delivery Outcomes and Body Composition

Birth weight and gestational age were obtained from obstetric maternity records. Gestational age was calculated from a 10- to 12-week ultrasound dating scan and date of birth. Venous cord-blood samples were obtained at delivery. Following < 12 h refrigeration at 4 °C in EDTA tubes, samples were spun, frozen and stored at − 80 °C. After transit to the Biochemistry Department of Glasgow Royal Infirmary, concentrations of cord-blood leptin were estimated by a highly sensitive and validated in-house ELISA that exhibits better sensitivity at lower levels than commercial assays [35]. Cord-blood insulin was measured by an ultrasensitive solid-phase two-site immunoassay ELISA (Mercodia, Uppsala, Sweden) that does not cross-react with proinsulin. Cord bloods were analysed in two batches, in 2012 and in 2017. Within 24–72 h of delivery, abdominal (around the umbilicus) and mid-upper arm circumferences were measured as part of routine neonatal examinations. These data were supplemented by research measurements of neonatal triceps and subscapular skinfolds. Routinely collected measurements were taken by trained paediatricians and midwives, whereas research measures (skinfolds) were taken by specially trained research assistants, all have been shown to provide reliable anthropometrical data [36].

### Covariates

Maternal date of birth (used to calculate age at recruitment), parity (number of children previously given birth to), and family history of high blood pressure and diabetes were obtained from antenatal medical records. Gestational age at recording of maternal measurements was derived from the 10- to 12-week ultrasound dating scan and the date of measurements. A latent multidimensional socioeconomic status (SES) indicator was synthesised from participant reports about their own (and if applicable their partner’s) education, employment status, housing tenure, financial situation, and ownership of material items and goods [37]. Select items from the 28-item General Health Questionnaire [38] were used; specifically components asking women if they had (1) been feeling perfectly well and in good health, and (2) been able to enjoy their normal day-to-day activities, were combined to a single categorical variable. To identify sleep problems, items about whether women had (1) lost much sleep over worry, and (2) had difficulty staying asleep, were also combined. Women were further grouped according to whether they had smoked tobacco cigarettes at any stage during pregnancy or the three months before, drank alcohol in pregnancy or in the three months before, consumed > 200 mg/day of caffeine [39], and had taken any dietary supplements such as vitamins or iron tablets in the past month (information regarding specific supplements was unavailable). Maternal weight (at 10–12 weeks gestation) was abstracted from antenatal medical records and combined with height measured at recruitment to derive early-pregnancy body mass index (BMI, kg/m^2^). Route of delivery (vaginal or caesarean), child sex and gestational age at birth were obtained from obstetric medical records.

### Descriptive Statistics

Participant characteristics were summarised by ethnic group and physical activity level. Linear and logistic regression trend-tests were used to explore differences in covariates across physical activity categories. Regression was further used to compare the ethnic and socioeconomic composition, and the distribution of maternal early-pregnancy BMI, between the analytical sample of this study against all excluded white British and Pakistani-origin women (*n *= 4814). Robust standard errors were used to account for clustered data from 384 women who provided information for more than one pregnancy.

### Main Analysis

To allow for missing data in covariates for 1127 participants (15.4% of the sample), multiple imputation by chained equations was used to create 20 complete datasets under the missing-at-random (MAR) assumption [40]. The imputation procedure included all variables and imputed outcomes were deleted prior to the main analysis [41].

Linear regression models were used to calculate adjusted differences (presented as estimated marginal means with 95% confidence intervals) in maternal and neonatal outcomes between the four groups of maternal mid-pregnancy physical activity (reference group: inactive). We also report *p* values from trend-tests across physical activity categories. A priori all analyses were stratified by maternal ethnicity (formal interaction tests confirmed effect modification between exposures and outcomes by ethnic group, *p *< 0.05) and were specified with robust standard errors to account for maternal clustering of data. When deemed necessary based on visual inspection of plots, outcomes were natural log transformed for analyses to improve normality of residuals. For logged outcomes, marginal means and confidence intervals were back-transformed for reporting via the exponentiation function.

#### Maternal Cardiometabolic Health

Following crude associations, preliminary regression models were adjusted for maternal age, gestational age at measurement, SES, and parity. Model 1 then further adjusted for lifestyle factors in pregnancy including smoking, alcohol, caffeine intake, sleep problems, and use of diet supplements. Model 2 was additionally adjusted for early-pregnancy BMI (except when triceps skinfold thickness and mid-upper arm circumference were modelled as outcomes).

#### Neonatal Delivery Outcomes and Body Composition

Statistical models were specified identically to maternal outcomes, but from the outset models were further adjusted for gestational age at birth (when itself not an outcome), child sex, and mode of delivery.

#### Sensitivity Analyses

For all outcomes, analyses were performed to adjust for physical activity reported during Ramadan [42], measurement season, and if women had been feeling well and able to enjoy their normal daily activities at the time of reporting. Adjustment for maternal family history of high blood pressure and diabetes was also carried out, as was whether Pakistani-origin women and their partners were born in Pakistan or the UK. The latter attempted to control for lifestyle and health-related pregnancy characteristics related to acculturation or Westernisation [43]. We also adjusted for the year of cord blood analysis to correct for potential batch effects. All analyses were performed in Stata/SE version 15.1 software.

## Results

### Participant Characteristics

Descriptive statistics for the non-imputed sample are presented in Tables 1 and 2. More than half of all women (57.7%), and a higher proportion of Pakistani-origin (73.7%) than white British women (39.2%), were inactive in mid-pregnancy. Approximately one-fifth of white British women reported data which categorised them as somewhat active (23.9%) and moderately active (21.1%), respectively; fewer were classified as active (15.8%). A smaller proportion of Pakistani-origin than white British women reported they were somewhat active (13.9%), moderately active (6.3%) and active (6.1%), respectively. The data were equally distributed across seasons (approximately one-quarter of records were from spring, summer, autumn, and winter) and < 10% of physical activity observations were reported during Ramadan.

**Table 1:** Characteristics of study participants: continuous variables

	Participants: observations (*n* in each activity category)	Inactive	Somewhat active	Moderately active	Active	*p* trend
White British						
Maternal age (years)	3263: 3400 (1334/812/718/536)	**25 (9)**	**26 (9)**	**27 (8)**	**27 (8.5)**	**<0.001**
Gestational age at recruitment (weeks)	3259: 3395 (1331/811/717/536)	**26.8 ± 2.2**	**26.5 ± 1.7**	**26.6 ± 1.7**	**26.5 ± 2.1**	**0.017**
Maternal early-pregnancy BMI (kg/m^2^)	3157: 3278 (1289/781/696/512)	25.6 (7.8)	25.6 (7.9)	25.8 (8.1)	24.7 (6.9)	0.14
Pakistani-origin						
Maternal age (years)	3658: 3905 (2880/543/245/237)	27 (7)	27 (7)	27 (6)	28 (7)	0.80
Gestational age at recruitment (weeks)	3655: 3901 (2876/543/245/237)	26.6 ± 2.1	26.9 ± 2.0	26.6 ± 2.1	26.6 ± 1.9	0.61
Maternal early-pregnancy BMI (kg/m^2^)	3518: 3747 (2755/526/240/226)	24.6 (6.8)	25.0 (6.9)	25.6 (7.2)	25.2 (6.5)	0.087

Values are mean ± standard deviation or median (interquartile range) given skewness. Bold font denotes significantly different values across physical activity categories (*p* trend < 0.05 from linear regression analysis, skewed variables natural log transformed prior to analysis)

**Table 2:** Characteristics of study participants: categorical variables

	Participants: observations (*n* in each activity category)	Inactive	Somewhat active	Moderately active	Active	*p* trend
White British						
Socioeconomic status	3260: 3396 (1334/809/718/535)					
Least deprived most educated		**128 (9.6)**	**126 (15.6)**	**140 (19.5)**	**117 (21.9)**	
Employed not materially deprived		**346 (25.9)**	**294 (36.3)**	**277 (38.6)**	**180 (33.6)**	
Employed no access to money		**180 (13.5)**	**146 (18.1)**	**127 (17.7)**	**81 (15.1)**	
Benefits but coping		**298 (22.3)**	**128 (15.8)**	**84 (11.7)**	**72 (13.5)**	
Most deprived		**382 (28.6)**	**115 (14.2)**	**90 (12.5)**	**85 (15.9)**	**<0.001**
Parity	3221: 3354 (1313/803/709/529)					
0		**569 (43.3)**	**423 (52.7)**	**355 (50.1)**	**258 (48.8)**	
1		**422 (32.1)**	**249 (31.0)**	**230 (32.4)**	**167 (31.6)**	
≥ 2		**322 (24.5)**	**131 (16.3)**	**124 (17.5)**	**104 (19.7)**	**0.001**
Smoked in pregnancy or 3 months before	3261: 3398 (1333/811/718/536)	**566 (42.5)**	**256 (31.6)**	**214 (29.8)**	**159 (29.7)**	**<0.001**
Consumed alcohol in pregnancy or 3 months before	3258: 3395 (1332/810/718/535)	891 (66.9)	524 (64.7)	459 (63.9)	354 (66.2)	0.45
Caffeine intake > 200 mg/day	3257: 3391 (1329/811/715/536)	**326 (24.5)**	**164 (20.2)**	**128 (17.9)**	**105 (19.6)**	**0.001**
Sleep problems	3225: 3358 (1298/811/716/533)					
No		558 (43.0)	356 (43.9)	313 (43.7)	228 (42.8)	
Lost or broken sleep		458 (35.3)	297 (36.6)	256 (35.8)	190 (35.6)	
Lost and broken sleep		282 (21.7)	158 (19.5)	147 (20.5)	115 (21.6)	0.90
Using dietary supplements	3262: 3399 (1333/812/718/536)	**361 (27.1)**	**225 (27.7)**	**242 (33.7)**	**197 (36.8)**	**<0.001**
Feel well and able to enjoy normal daily activities	3228: 3363 (1301/811/716/535)					
Yes		717 (55.1)	455 (56.1)	407 (56.8)	300 (56.1)	
Somewhat		362 (27.8)	238 (29.3)	211 (29.5)	158 (29.5)	
No		222 (17.1)	118 (14.6)	98 (13.7)	77 (14.4)	0.28
Maternal family history of hypertension	3090: 3215 (1261/769/681/504)	282 (22.4)	205 (26.7)	169 (24.8)	119 (23.6)	0.41
Maternal family history of diabetes	3086: 3210 (1258/770/679/503)	184 (14.6)	109 (14.2)	93 (13.7)	65 (12.9)	0.33
Mode of delivery	3263: 3400 (1334/812/718/536)					
Vaginal		**1058 (79.3)**	**630 (77.6)**	**549 (76.5)**	**400 (74.6)**	**0.020**
Caesarean		**276 (20.7)**	**182 (22.4)**	**169 (23.5)**	**136 (25.4)**	
Offspring sex	3263: 3400 (1334/812/718/536)					
Male		669 (50.1)	425 (52.3)	383 (53.3)	279 (52.0)	0.26
Female		665 (49.9)	387 (47.7)	335 (46.7)	257 (48.0)	
Pakistani-origin						
Socioeconomic status	3652: 3898 (2875/541/245/237)					
Least deprived most educated		**462 (16.1)**	**140 (25.9)**	**71 (29.0)**	**63 (26.6)**	
Employed not materially deprived		**139 (4.8)**	**112 (20.7)**	**62 (25.3)**	**41 (17.3)**	
Employed no access to money		**418 (14.5)**	**98 (18.1)**	**35 (14.3)**	**37 (15.6)**	
Benefits but coping		**1424 (49.5)**	**154 (28.5)**	**66 (26.9)**	**72 (30.4)**	
Most deprived		**432 (15.0)**	**37 (6.8)**	**11 (4.5)**	**24 (10.1)**	**<0.001**
Parity	3608: 3845 (2838/536/237/234)					
0		**831 (29.3)**	**219 (40.9)**	**98 (41.4)**	**94 (40.2)**	
1		**752 (26.5)**	**159 (29.7)**	**69 (29.1)**	**52 (22.2)**	
≥ 2		**1255 (44.2)**	**158 (29.5)**	**70 (29.5)**	**88 (37.6)**	**<0.001**
Smoked in pregnancy or 3 months before	3650: 3895 (2870/543/245/237)	**89 (3.1)**	**22 (4.1)**	**18 (7.4)**	**12 (5.1)**	**0.002**
Consumed alcohol in pregnancy or 3 months before	3650: 3895 (2870/543/245/237)	7 (0.2)	1 (0.2)	4 (1.6)	0 (0)	0.16
Caffeine intake > 200 mg/day	3644: 3889 (2864/543/245/237)	187 (6.5)	30 (5.5)	13 (5.3)	24 (10.1)	0.31
Sleep problems	3441: 3640 (2621/538/244/237)					
No		1353 (51.6)	272 (50.6)	111 (45.5)	116 (48.9)	
Lost or broken sleep		674 (25.7)	121 (22.5)	75 (30.7)	58 (24.5)	
Lost and broken sleep		594 (22.7)	145 (27.0)	58 (23.8)	63 (26.6)	0.070
Using dietary supplements	3645: 3890 (2866/543/245/236)	1367 (47.7)	271 (49.9)	127 (51.8)	111 (47.0)	0.50
Feel well and able to enjoy normal daily activities	3438: 3636 (2618/538/244/236)					
Yes		**1346 (51.4)**	**275 (51.1)**	**121 (49.6)**	**106 (44.9)**	
Somewhat		**895 (34.2)**	**151 (28.1)**	**79 (32.4)**	**83 (35.2)**	
No		**377 (14.4)**	**112 (20.8)**	**44 (18.0)**	**47 (19.9)**	**0.012**
Maternal family history of hypertension	3454: 3672 (2704/515/227/226)	**797 (29.5)**	**177 (34.4)**	**83 (36.6)**	**77 (34.1)**	**0.007**
Maternal family history of diabetes	3448: 3670 (2702/516/226/226)	**922 (34.1)**	**226 (43.8)**	**104 (46.0)**	**95 (42.0)**	**<0.001**
Mode of delivery	3658: 3904 (2879/543/245/237)					
Vaginal		**2328 (80.9)**	**431 (79.4)**	**174 (71.0)**	**178 (75.1)**	**<0.001**
Caesarean		**551 (19.1)**	**112 (20.6)**	**71 (29.0)**	**59 (24.9)**	
Offspring sex	3658: 3904 (2879/543/245/237)					
Male		1452 (50.4)	276 (50.8)	129 (52.7)	131 (55.3)	0.15
Female		1427 (49.6)	267 (49.2)	116 (47.3)	106 (44.7)	
Country of birth	3569: 3807 (2811/530/236/230)					
Mother and partner born in Pakistan		**984 (35.0)**	**106 (20.0)**	**34 (14.4)**	**43 (18.7)**	
Only partner born in UK		**878 (31.2)**	**56 (10.6)**	**25 (10.6)**	**40 (17.4)**	
Only mother born in UK		**668 (23.8)**	**246 (46.4)**	**120 (50.8)**	**98 (42.6)**	
Mother and partner born in UK		**281 (10.0)**	**122 (23.0)**	**57 (24.2)**	**49 (21.3)**	**<0.001**

Values are *n* (%). Bold font denotes significantly different values across physical activity categories (*p* trend < 0.05 from logistic regression analyses for 2-level categories and ordered logistic regression for > 2-level categories). In white British women, when adjusted for socioeconomic status *p* trend = 0.19 for mode of delivery, the results for Pakistani-origin women were unchanged

Trend-tests revealed that women who reported themselves to be more active mid-pregnancy were less deprived, more likely to be nulliparous, and were more likely to deliver by caesarean section (although this difference did not persist in white British women when delivery mode was adjusted for SES, *p *= 0.19). White British women who were more active were also older, more likely to take dietary supplements, and were less likely to both smoke in pregnancy and consume > 200 mg/day of caffeine. Pakistani-origin women who were more active in mid-pregnancy were more likely to have been born in the UK, smoke in pregnancy, and have a family history of hypertension and diabetes. The analytical sample of this study did not differ from excluded white British and Pakistani-origin women in respect to ethnic and socioeconomic composition, or the distribution of maternal early-pregnancy BMI.

### Maternal Cardiometabolic Health

**Fig. 1:**
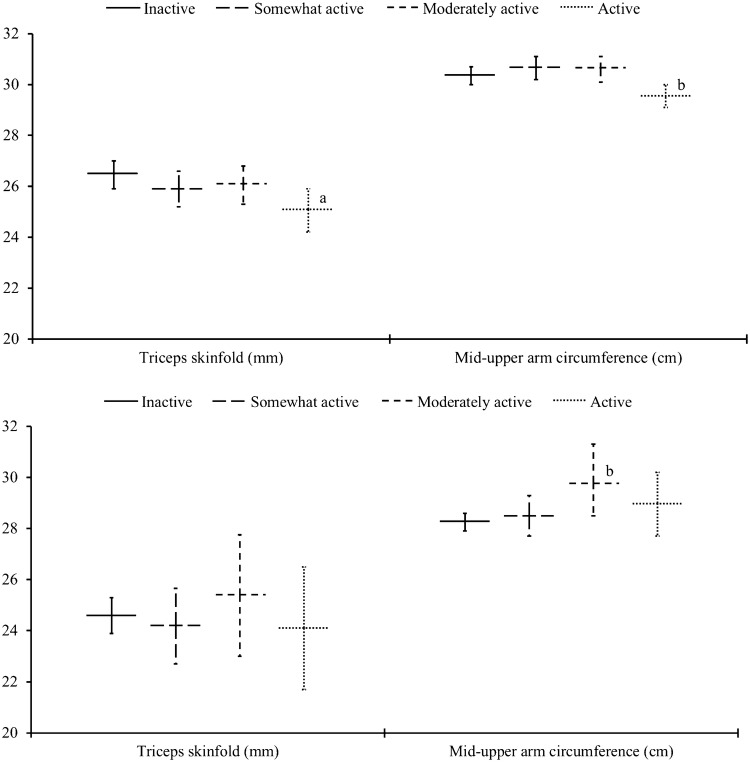
Associations of mid-pregnancy physical activity with maternal adiposity. The top tile relates to white British women and the bottom tile Pakistani-origin women. Data are estimated marginal means (95% confidence interval) adjusted for maternal age, gestational age at measurement, socioeconomic status, parity, maternal smoking, alcohol consumption, caffeine intake, sleep quality, and use of dietary supplements. ‘a’ (*p *< 0.01) and ‘b’ (*p *< 0.05) denote significantly different values compared to the referent inactive group. *p* trend = 0.016 across physical activity categories for triceps skinfold in white British women. Number of participants (observations): white British women (Triceps skinfold: *n *= 1662 (1700); Mid-upper arm circumference: *n *= 1673 (1712)), Pakistani-origin women (Triceps skinfold: *n *= 585 (590); Mid-upper arm circumference: *n *= 590 (595)). *n* in each activity category for white British women: Inactive (Triceps skinfold: *n *= 694; Mid-upper arm circumference: *n *= 700), Somewhat active (Triceps skinfold: *n *= 405; Mid-upper arm circumference: *n *= 409), Moderately active (Triceps skinfold: *n *= 346; Mid-upper arm circumference: *n *= 348), Active (Triceps skinfold: *n *= 255; Mid-upper arm circumference: *n *= 255). *n* in each activity category for Pakistani-origin women: Inactive (Triceps skinfold: *n *= 424; Mid-upper arm circumference: *n *= 427), Somewhat active (Triceps skinfold: *n *= 88; Mid-upper arm circumference: *n *= 88), Moderately active (Triceps skinfold: *n *= 41; Mid-upper arm circumference: *n *= 42), Active (Triceps skinfold: *n *= 37; Mid-upper arm circumference: *n *= 38). See Supplementary File 2 for full results

**Fig. 2:**
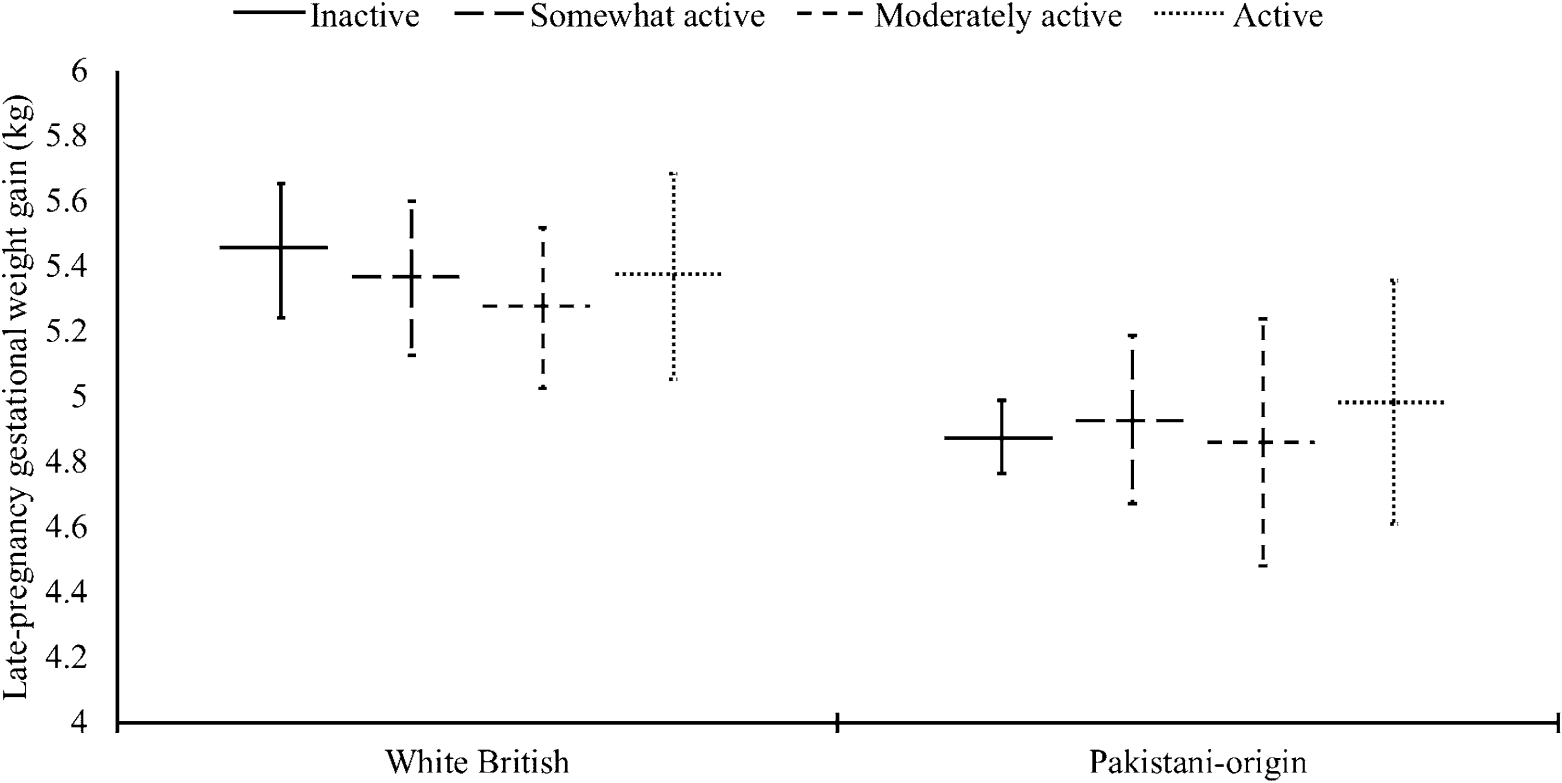
Associations of mid-pregnancy physical activity with late-pregnancy gestational weight gain. Data are estimated marginal means (95% confidence interval) adjusted for maternal age, gestational age at measurement, socioeconomic status, parity, maternal smoking, alcohol consumption, caffeine intake, sleep quality, use of dietary supplements, maternal early-pregnancy BMI, and the number of weeks between mid- and late- pregnancy weight measurements. Number of participants (observations) for white British women: 1120 (1137), and Pakistani-origin women 1582 (1628). *n* in each activity category for white British women: Inactive (*n *= 424), Somewhat active (*n *= 286), Moderately active (*n *= 260), Active (*n *= 167). *n* in each activity category for Pakistani-origin women: Inactive (*n *= 1181), Somewhat active (*n *= 245), Moderately active (*n *= 92), Active (*n *= 110). See Supplementary File 2 for full results

Figure 1 shows levels of maternal pregnancy adiposity stratified by physical activity categories. In white British women, there was a significant trend showing that more mid-pregnancy physical activity was associated with smaller triceps skinfold thickness. White British women who were categorised as active were consistently less adipose, they had significantly smaller triceps skinfolds and a smaller mid-upper arm circumference compared to white British women who were inactive. Pakistani-origin women who were moderately active had a larger mid-upper arm circumference compared to Pakistani-origin women who were inactive (see Supplementary File 2 for full results). No associations were found between mid-pregnancy physical activity and late-pregnancy gestational weight gain (Fig. 2).

Table 3 shows there was no evidence for associations of mid-pregnancy physical activity with insulin or glucose concentrations in Pakistani-origin women. However, Pakistani-origin women who were somewhat active had slightly lower systolic blood pressure than their inactive counterparts. In white British women there were significant trends showing that more mid-pregnancy physical activity was associated with lower fasting insulin and postload glucose concentrations; values were consistently lower in white British women who were categorised as active. Table 4 shows there was also a significant trend that more mid-pregnancy physical activity was associated with lower triglyceride levels in white British women; lower values were seen in moderately active and active white British women compared to inactive white British women. Pakistani-origin women who were somewhat active exhibited lower triglyceride concentrations, in addition to higher high-density lipoprotein cholesterol (HDL-c) levels and a lower ratio of total to HDL-c, compared to Pakistani-origin women who were inactive.

**Table 3:** Associations of mid-pregnancy physical activity with maternal insulin and glucose, and blood pressure

	Participants: observations (*n* in each activity category)	Inactive	Somewhat active	Moderately active	Active	*p* trend
White British						
Fasting insulin (pmol/l)	3099: 3225 (1256/771/688/510)	71.8 (70.1–73.6) Ref	72.4 (70.2–74.7) 0.68	70.1 (67.9–72.3) 0.22	**68.2 (65.9**–**70.6)** **0.017**	**0.014**
Fasting glucose (mmol/l)	3145: 3273 (1282/787/688/516)	4.4 (4.3–4.4) Ref	4.4 (4.3–4.4) 0.68	4.4 (4.3–4.4) 0.29	4.4 (4.3–4.4) 0.92	0.67
Postload glucose (mmol/l)	3142: 3270 (1280/787/687/516)	5.4 (5.3–5.4) Ref	5.3 (5.2–5.4) 0.17	5.3 (5.2–5.4) 0.050	**5.2 (5.1**–**5.3)** **0.012**	**0.006**
Systole (mmHg)	3173: 3302 (1284/792/701/525)	112 (112–113) Ref	112 (111–113) 0.41	112 (111–113) 0.68	112 (111–113) 0.41	0.47
Diastole (mmHg)	3173: 3303 (1285/792/701/525)	66 (66–67) Ref	66 (65–66) 0.25	66 (65–66) 0.32	66 (65–67) 0.61	0.46
Pakistani-origin						
Fasting insulin (pmol/l)	3281: 3478 (2564/481/219/214)	87.0 (85.4–88.6) Ref	85.3 (81.7–89.1) 0.42	84.2 (79.7–89.0) 0.28	84.9 (79.9–90.3) 0.46	0.23
Fasting glucose (mmol/l)	3547: 3783 (2789/526/237/231)	4.6 (4.5–4.6) Ref	4.6 (4.5–4.6) 0.59	4.6 (4.5–4.6) 0.76	4.6 (4.5–4.6) 0.81	0.83
Postload glucose (mmol/l)	3545: 3780 (2787/525/237/231)	5.7 (5.6–5.7) Ref	5.6 (5.5–5.7) 0.29	5.5 (5.4–5.7) 0.13	5.6 (5.5–5.8) 0.63	0.20
Systole (mmHg)	3522: 3750 (2762/523/235/230)	107 (107–108) Ref	**106 (105**–**107)** **0.047**	107 (105–108) 0.58	108 (106–109) 0.37	0.99
Diastole (mmHg)	3522: 3750 (2762/523/235/230)	64 (63–64) Ref	63 (62–64) 0.095	64 (63–65) 0.71	64 (63–65) 0.87	0.69

Data are estimated marginal means (95% confidence interval) adjusted for maternal age, gestational age at measurement, socioeconomic status, parity, maternal smoking, alcohol consumption, caffeine intake, sleep quality, use of dietary supplements, and maternal early-pregnancy BMI. Below the estimates are *p*-values. Bold font denotes significantly different values compared to the referent inactive group (*p *< 0.05) or across physical activity categories (*p* trend < 0.05)

**Table 4:** Associations of mid-pregnancy physical activity with maternal lipid and lipoprotein cholesterol

	Participants: observations (*n* in each activity category)	Inactive	Somewhat active	Moderately active	Active	*p* trend
White British						
Total cholesterol (mmol/l)	3098: 3224 (1255/771/688/510)	6.4 (6.3–6.5) Ref	6.5 (6.4–6.5) 0.39	6.4 (6.3–6.5) 0.73	6.4 (6.3–6.5) 0.75	0.62
Triglycerides (mmol/l)	3098: 3224 (1255/771/688/510)	1.9 (1.9–2.0) Ref	1.9 (1.8–1.9) 0.23	**1.8 (1.8**–**1.9)** **0.003**	**1.8 (1.8**–**1.9)** **0.003**	**< 0.001**
LDL cholesterol (mmol/l)	3075: 3200 (1242/766/684/508)	3.5 (3.5–3.6) Ref	3.6 (3.5–3.7) 0.14	3.6 (3.5–3.6) 0.50	3.5 (3.4–3.6) 0.82	0.76
HDL cholesterol (mmol/l)	3098: 3224 (1255/771/688/510)	2.0 (1.9–2.0) Ref	2.0 (1.9–2.0) 0.85	2.0 (1.9–2.0) 0.69	2.0 (2.0–2.1) 0.33	0.52
Total: HDL ratio	3098: 3224 (1255/771/688/510)	3.3 (3.3–3.4) Ref	3.3 (3.3–3.4) 0.66	3.3 (3.3–3.4) 0.95	3.3 (3.2–3.3) 0.23	0.33
Pakistani-origin						
Total cholesterol (mmol/l)	3283: 3480 (2566/481/219/214)	6.0 (6.0–6.1) Ref	6.0 (5.9–6.1) 0.80	6.0 (5.9–6.1) 0.68	6.1 (5.9–6.2) 0.82	0.95
Triglycerides (mmol/l)	3283: 3480 (2566/481/219/214)	1.9 (1.8–1.9) Ref	**1.8 (1.7**–**1.9)** **0.040**	1.8 (1.7–1.9) 0.20	1.8 (1.7–1.9) 0.49	0.13
LDL cholesterol (mmol/l)	3262: 3456 (2549/477/218/212)	3.2 (3.1–3.2) Ref	3.2 (3.1–3.3) 0.52	3.2 (3.0–3.3) 0.60	3.2 (3.1–3.3) 0.84	0.77
HDL cholesterol (mmol/l)	3283: 3480 (2566/481/219/214)	1.9 (1.9–2.0) Ref	**2.0 (2.0**–**2.1)** **0.014**	2.0 (1.9–2.0) 0.37	2.0 (1.9–2.1) 0.45	0.15
Total :HDL ratio	3283: 3480 (2566/481/219/214)	3.2 (3.1–3.2) Ref	**3.1 (3.0**–**3.1)** **0.012**	3.1 (3.0–3.2) 0.11	3.1 (3.1–3.2) 0.70	0.12

Data are estimated marginal means (95% confidence interval) adjusted for maternal age, gestational age at measurement, socioeconomic status, parity, maternal smoking, alcohol consumption, caffeine intake, sleep quality, use of dietary supplements, and maternal early-pregnancy BMI. Below the estimates are *p*-values. Bold font denotes significantly different values compared to the referent inactive group (*p *< 0.05) or across physical activity categories (*p* trend < 0.05)

### Neonatal Delivery Outcomes and Body Composition

Table 5 shows that in white British women there were significant trends indicating that more mid-pregnancy physical activity was associated with smaller offspring triceps and subscapular skinfolds; lower values for both sites were seen in moderately active and active white British women compared to inactive white British women. Babies of Pakistani-origin women who were somewhat active had smaller abdominal and mid-upper arm circumferences, and a lower cord-blood leptin concentration, compared to babies of Pakistani-origin women who were inactive.

**Table 5:** Associations of mid-pregnancy physical activity with gestational age at birth, birth weight and offspring adiposity

	Participants: observations (*n* in each activity category)	Inactive	Somewhat active	Moderately active	Active	*p* trend
White British						
Gestational age (months)	3263: 3400 (1334/812/718/536)	39.6 (39.5–39.7) Ref	39.7 (39.5–39.8) 0.26	39.6 (39.4–39.7) 0.83	39.7 (39.5–39.8) 0.19	0.32
Birth weight (g)	3262: 3399 (1334/812/717/536)	3374 (3350–3397) Ref	3346 (3317–3375) 0.15	3369 (3338–3400) 0.83	3366 (3329–3402) 0.71	0.80
Sum of skinfolds (mm)	2275: 2333 (938/566/468/361)	10.2 (10.0–10.3) Ref	10.1 (9.9–10.2) 0.40	**9.9 (9.7**–**10.0)** **0.010**	**9.9 (9.7**–**10.1)** **0.038**	**0.007**
Triceps skinfold (mm)	2284: 2342 (943/567/470/362)	5.3 (5.2–5.3) Ref	5.2 (5.1–5.3) 0.87	5.1 (5.0–5.2) 0.071	5.1 (5.0–5.2) 0.094	**0.034**
Subscapular skinfold (mm)	2276: 2334 (939/566/468/361)	4.9 (4.8–5.0) Ref	4.8 (4.7–4.9) 0.14	**4.7 (4.6**–**4.8)** **0.002**	**4.8 (4.7**–**4.8)** **0.032**	**0.004**
Abdominal circumference (cm)	2879: 2965 (1174/714/608/469)	32.0 (31.8–32.1) Ref	31.9 (31.7–32.1) 0.65	32.1 (31.9–32.3) 0.27	32.0 (31.7–32.2) 0.86	0.70
Mid-upper arm circumference (cm)	2882: 2967 (1175/714/608/470)	10.9 (10.8–10.9) Ref	10.8 (10.7–10.8) 0.063	10.8 (10.7–10.9) 0.16	10.8 (10.7–10.9) 0.098	0.062
Cord leptin (ng/ml)	1943: 1995 (808/466/422/299)	6.6 (6.2–7.0) Ref	6.5 (6.0–7.0) 0.80	6.7 (6.2–7.3) 0.61	6.5 (5.9–7.2) 0.88	0.89
Cord insulin (pmol/l)	1936: 1987 (806/464/420/297)	3.6 (3.4–3.8) Ref	3.6 (3.3–3.8) 0.86	3.5 (3.2–3.8) 0.50	3.5 (3.1–3.8) 0.52	0.43
Pakistani-origin						
Gestational age (months)	3658: 3905 (2880/543/245/237)	39.4 (39.3–39.5) Ref	39.4 (39.2–39.6) 0.88	39.5 (39.2–39.7) 0.81	39.5 (39.3–39.8) 0.42	0.51
Birth weight (g)	3658: 3905 (2880/543/245/237)	3145 (3130–3161) Ref	3114 (3080–3148) 0.11	3155 (3097–3213) 0.76	3142 (3094–3190) 0.91	0.78
Sum of skinfolds (mm)	2817: 2926 (2176/398/174/178)	9.6 (9.5–9.7) Ref	9.6 (9.4–9.8) 0.57	9.7 (9.4–10.0) 0.52	9.7 (9.4–9.9) 0.71	0.65
Triceps skinfold (mm)	2827: 2936 (2184/399/175/178)	5.0 (5.0–5.1) Ref	5.0 (4.9–5.1) 0.45	5.1 (4.9–5.2) 0.61	5.0 (4.9–5.1) 0.90	0.98
Subscapular skinfold (mm)	2819: 2928 (2177/399/174/178)	4.6 (4.6–4.7) Ref	4.6 (4.5–4.7) 0.77	4.7 (4.5–4.8) 0.46	4.7 (4.5–4.8) 0.45	0.39
Abdominal circumference (cm)	3237: 3404 (2519/473/210/202)	30.7 (30.6–30.8) Ref	**30.4 (30.2**–**30.6)** **0.043**	30.3 (29.9–30.7) 0.070	30.7 (30.4–31.0) 0.93	0.22
Mid-upper arm circumference (cm)	3230: 3396 (2512/473/209/202)	10.6 (10.5–10.6) Ref	**10.4 (10.3**–**10.5)** **0.030**	10.5 (10.4–10.7) 0.59	10.5 (10.4–10.6) 0.49	0.21
Cord leptin (ng/ml)	2234: 2315 (1721/309/149/136)	7.6 (7.3–7.9) Ref	**6.4 (5.9**–**7.1)** **0.002**	6.9 (6.0–7.9) 0.23	7.1 (6.1–8.3) 0.48	0.083
Cord insulin (pmol/l)	2216: 2295 (1707/308/147/133)	4.1 (3.9–4.2) Ref	3.9 (3.6–4.3) 0.49	4.0 (3.5–4.5) 0.75	3.8 (3.3–4.4) 0.42	0.36

Data are estimated marginal means (95% confidence interval) adjusted for maternal age, gestational age at measurement, socioeconomic status, parity, gestational age (when itself was not an outcome), child sex, mode of delivery, maternal smoking, alcohol consumption, caffeine intake, sleep quality, use of dietary supplements, and maternal early-pregnancy BMI. Below the estimates are *p*-values. Bold font denotes significantly different values compared to the referent inactive group (*p *< 0.05) or across physical activity categories (*p* trend < 0.05)

### Sensitivity Analyses

All results were fundamentally unchanged in sensitivity and complete-case analyses, but in the latter confidence intervals expectedly widened (see Supplementary File 2).

## Discussion

In this observational study, physical activity performed mid-pregnancy was associated with several cardiometabolic health markers in both white British and Pakistani-origin women. The results indicate that physical activity may help to attenuate pregnancy-related insulin resistance and dyslipidaemia. Our associations further imply that women who are more active mid-pregnancy may have children with lower adiposity at birth, without adversely influencing birth weight or gestational age. Some important differences were noted between ethnic groups. Dose-dependent associations were consistently observed in white British women, suggesting better metabolic health with higher levels of mid-pregnancy physical activity. Indeed, white British women who reported themselves to be active, and who therefore broadly met the recommended 150 min of moderate intensity physical activity per week, were metabolically healthiest in terms of the markers studied here. In Pakistani-origin women, a threshold-type effect was seen, with better cardiometabolic health only in Pakistani-origin women who were somewhat active. This could be explained by small sample size and limited statistical power at the higher end of the activity spectrum, because few women of Pakistani-origin were categorised as moderately active and active, respectively. It is nonetheless encouraging that even a small volume of physical activity, albeit below the recommended level, appeared to confer some cardiometabolic benefit to Pakistani-origin women and their offspring. UK guidelines emphasise that ‘every activity counts’ and that inactive pregnant women should gradually accumulate physical activity throughout the week [14]. This advice appears highly relevant to Pakistani-origin women, for whom modest increases in mid-pregnancy physical activity could translate to population-level changes in cardiometabolic health, helping to reduce health inequality.

### Maternal Cardiometabolic Health

Meta-analyses of experimental and cohort studies indicate that more active women gain less weight during pregnancy and are at lower risk of excess gestational weight gain [10]. We found no associations between mid-pregnancy physical activity with subsequent weight gain in either ethnic group. This might be because we were only able to investigate late-pregnancy gestational weight gain. We did find that white British women who were active had, on average, smaller triceps skinfold thickness and mid-upper arm circumferences compared to white British women who were inactive. Counterintuitively, Pakistani-origin women who were moderately active exhibited larger mid-upper arm circumferences than Pakistani-origin women who were inactive. This particular observation, however, should be interpreted with caution due to probable selection bias. Only a small group of Pakistani-origin women (~ 15%) provided a triceps skinfold and arm circumference measurement, and those who did were less deprived (*p *< 0.001) and had lower early-pregnancy BMI compared to Pakistani-origin women who opted out of these measurements (24.4 vs. 25.7 kg/m^2^, *p *< 0.001).

Recent meta-analyses have indicated that pregnancy physical activity is associated with ~ 20–35% reduced risk of abnormal glucose tolerance (within the non-diabetic range), gestational diabetes, and gestational hypertension, respectively [3, 44]. An individual participant meta-analysis of experimental data concluded that effects were consistent across ethnic groups, but the number of non-white mothers was small, ethnicity was broadly classified as white or not, and the majority of participants were highly educated [3]. Independently of covariates, including early-pregnancy BMI, we found that white British women who were active exhibited lower fasting insulin and postload glucose levels than white British women who were inactive. There was no evidence for equivalent associations in Pakistani-origin women, other than in complete-case analysis a weak trend toward lower postload glucose levels with more mid-pregnancy physical activity (*p* trend = 0.094; see Supplementary File 2). The ethnic difference in associations may be real, but equally too few Pakistani-origin women may have been sufficiently active in terms of volume or intensity to permit detection of activity mediated improvements in insulin sensitivity and glycaemic control. The threshold level of physical activity needed to confer benefit may also be higher in Pakistani-origin women, as seems to be the case in the general South Asian population for reduced type 2 diabetes risk [45]. We found no associations for blood pressure in white British women. Pakistani-origin women who were somewhat active had, on average, lower systolic values than Pakistani-origin women who were inactive, but the difference was of limited clinical significance. Clinical decisions regarding antenatal care are based on several blood pressure measurements taken throughout pregnancy and blood pressure tends to increase as pregnancy progresses. Reliance on a single blood pressure measurement at 26–28 weeks gestation may have biased results toward the null.

Triglyceride levels were marginally lower in active than inactive white British women and in somewhat active than inactive Pakistani-origin women. The few studies conducted to date have also described small inverse associations for triglycerides, accompanied by reports of mostly null associations for total and lipoprotein cholesterol [7, 8]. It has been speculated that relatively small associations with lipoproteins may have gone undetected in previous studies due to low statistical power [46]. In our large biethnic cohort, HDL-c levels were on average slightly higher in Pakistani-origin women who were somewhat active than inactive, but no corresponding associations were found in white British women, and there was no evidence for associations with low-density lipoprotein cholesterol (LDL-c) levels in either ethnic group. Our results suggest that observing large samples may be required to detect small associations with blood lipid levels. Alternatively, favouring precise physical activity ascertainment over self-reported data, which are known to be subject to considerable error and biases, will reduce the sample size needed for a given statistical power. In only 206 pregnant women, Loprinzi et al. found that accelerometer-determined sedentary time was associated with higher LDL-c levels, and moderate-to-vigorous intensity physical activity predicted higher HDL-c concentrations [47]. Future studies should prioritise objective monitoring of physical activity. Wrist-worn accelerometry has the advantage of being well-tolerated by pregnant women and can be used in large study populations [48].

### Neonatal Delivery Outcomes and Body Composition

In white British women, there was a dose-dependent association between higher mid-pregnancy physical activity and smaller neonatal skinfolds. Pakistani-origin women who were somewhat active also had babies with, on average, smaller abdominal and mid-upper arm circumferences compared to Pakistani-origin women who were inactive. The differences were small, and may not be clinically meaningful, but could be influential with respect to future health and obesity risk. Moderate physical activity performed in pregnancy has recently been reported to reduce the odds of offspring obesity by 23% at age 8 years [49]. Crucially, we found that cord-blood leptin concentration (a valid biomarker of birth total fat mass [50]) was markedly lower in Pakistani-origin women who were somewhat active as opposed to inactive. Studies using lab-based techniques such as DXA and densitometry have likewise reported lower fat mass and body fat percentage in babies born to women who were more active during pregnancy [51, 52]. In line with other large-scale population-based studies [53, 54] and smaller studies incorporating accelerometry [55], we found no indication that physical activity adversely affected gestational age or birth weight. Recent meta-analyses indicate that leisure-time physical activity may be advantageous in reducing the risk of pre-term deliveries [9, 10] and large-for-gestational age births [10–12].

### Strengths and Limitations

This study was conducted in a large cohort of white British and Pakistani-origin women, allowing ethnicity-specific associations to be quantified for several maternal and neonatal health markers. We were unable to investigate additional South Asian groups due to limited numbers of participants with backgrounds other than in Pakistan. Focussing on Pakistani-origin women as opposed to a heterogeneous group of ‘South Asians’ (of Pakistani, Indian and Bangladeshi origin) reduced confounding potential, as did conducting the study in one economically deprived UK city, but both at the cost of generalisability. There is inherent error and bias in self-reported physical activity, the extent of which could have differed between ethnic groups, but we have no evidence of this. To ensure consistent meaning and interpretation of questions, irrespective of ethnicity, bilingual researchers administered questionnaires to all non-English speaking women. Presuming that any errors in self-reported physical activity were randomly distributed across levels of the exposure and outcomes, the magnitude of associations will have been diluted in both ethnic groups. This may in part explain the diminutive size of associations and requires consideration when adjudging clinical significance. Multiple imputation of missing covariates, and retaining all outcomes in their original (continuous) forms, optimised statistical power. Including clinical diagnoses as outcomes (e.g. gestational diabetes, gestational hypertension, prematurity, small- or large-for-gestational age) may have assisted in determining clinical relevance, but few cases in already small cells rendered models unstable. Residual confounding is probable due to error and bias in self-reported covariates, and unspecified confounding may exist, for example by dietary factors. We also relied upon a single report of physical activity at 26–28 weeks gestation. Future studies should gather repeated-measurements prior to pregnancy and throughout gestation, using objective methods to characterise relationships by physical activity volume and pattern, and with longer-term follow-up of outcomes.

## Conclusion

Mid-pregnancy physical activity was beneficially associated with maternal cardiometabolic health and neonatal adiposity in white British and Pakistani-origin women from a deprived urban setting, without influencing gestational age or birth weight. In white British women associations were dose-dependent, and women who reported themselves to be active (thus broadly meeting the recommended 150 min of moderate intensity physical activity per week) benefitted the most. In Pakistani-origin women, three-quarters of whom were inactive, even a small amount of mid-pregnancy physical activity appeared to benefit some markers of cardiometabolic health. Our results highlight the importance of modifiable risk factors in early life, and the potential for promoting physical activity in pregnancy to improve maternal and child health and reduce health inequalities.

## Electronic Supplementary Material

Below is the link to the electronic supplementary material.

Supplementary material 1 (DOCX 89 kb)

Supplementary material 2 (DOCX 33 kb)

## Data Availability

Scientists are encouraged and able to use the BiB data, and proposals for collaboration are welcomed. Information on how to access the data can be found at: www.borninbradford.nhs.uk. Guidance for researchers and collaborators, the study protocol and the data collection schedule are all available via the website. All requests are carefully considered and accepted when possible.

## References

[1] Davenport MH, Ruchat SM, Sobierajski F (2019). Impact of prenatal exercise on maternal harms, labour and delivery outcomes: a systematic review and meta-analysis. Br J Sports Med.

[2] Dipietro L, Evenson KR, Bloodgood B, Sprow K, Troiano RP, Piercy KL, Vaux-Bjerke A, Powell KE (2019). Benefits of physical activity during pregnancy and postpartum. Med Sci Sport Exerc.

[3] The International Weight Management in Pregnancy (i-WIP) Collaborative Group (2017). Effect of diet and physical activity based interventions in pregnancy on gestational weight gain and pregnancy outcomes: meta-analysis of individual participant data from randomised trials. BMJ.

[4] Damm P, Houshmand-Oeregaard A, Kelstrup L, Lauenborg J, Mathiesen ER, Clausen TD (2016). Gestational diabetes mellitus and long-term consequences for mother and offspring: a view from Denmark. Diabetologia.

[5] Catalano PM, Shankar K (2017). Obesity and pregnancy: mechanisms of short term and long term adverse consequences for mother and child. BMJ.

[6] Neiger R (2017). Long-term effects of pregnancy complications on maternal health: a review. J Clin Med.

[7] Mudd LM, Evenson KR (2015). Review of impacts of physical activity on maternal metabolic health during pregnancy. Curr Diab Rep.

[8] Boghossian NS, Orekoya O, Liu J, Liu J (2016). Pregnancy interventions or behaviors and cardiometabolic biomarkers: a systematic review. Curr Epidemiol Reports.

[9] Aune D, Schlesinger S, Henriksen T, Saugstad OD, Tonstad S (2017). Physical activity and the risk of preterm birth: a systematic review and meta-analysis of epidemiological studies. BJOG An Int J Obstet Gynaecol.

[10] da Silva SG, Ricardo LI, Evenson KR, Hallal PC (2017). Leisure-time physical activity in pregnancy and maternal-child health: a systematic review and meta-analysis of randomized controlled trials and cohort studies. Sport Med.

[11] Wiebe HW, Boulé NG, Chari R, Davenport MH (2015). The effect of supervised prenatal exercise on fetal growth. Obstet Gynecol.

[12] Pastorino S, Bishop T, Crozier SR (2019). Associations between maternal physical activity in early and late pregnancy and offspring birth size: remote federated individual level meta-analysis from eight cohort studies. BJOG An Int J Obstet Gynaecol.

[13] American College of Obstetricians and Gynecologists (2015). Physical activity and exercise during pregnancy and the postpartum period. Obstet Gynecol.

[14] Smith R, Reid H, Matthews A, Calderwood C, Knight M, Foster C (2018). Infographic: physical activity for pregnant women. Br J Sports Med.

[15] Mottola MF, Davenport MH, Ruchat SM (2018). 2019 Canadian guideline for physical activity throughout pregnancy. Br J Sports Med.

[16] Evenson KR, Savitz DA, Huston SL (2004). Leisure-time physical activity among pregnant women in the US. Paediatr Perinat Epidemiol.

[17] Borodulin KM, Evenson KR, Wen F, Herring AH, Benson AM (2008). Physical activity patterns during pregnancy. Med Sci Sports Exerc.

[18] Kraschnewski JL, Chuang CH, Downs DS, Weisman CS, McCamant EL, Baptiste-Roberts K, Zhu J, Kjerulff KH (2013). Association of prenatal physical activity and gestational weight gain: Results from the first baby study. Women’s Heal Issues.

[19] Nascimento SL, Surita FG, Godoy AC, Kasawara KT, Morais SS (2015). Physical activity patterns and factors related to exercise during pregnancy: a cross sectional study. PLoS One.

[20] Richardsen KR, Falk RS, Jenum AK, Mørkrid K, Martinsen EW, Ommundsen Y, Berntsen S (2016). Predicting who fails to meet the physical activity guideline in pregnancy: a prospective study of objectively recorded physical activity in a population-based multi-ethnic cohort. BMC Pregnancy Childbirth.

[21] Gaston A, Cramp A (2011). Exercise during pregnancy: a review of patterns and determinants. J Sci Med Sport.

[22] Evenson KR, Wen F (2011). Prevalence and correlates of objectively measured physical activity and sedentary behavior among US pregnant women. Prev Med (Baltim).

[23] Jenum AK, Mrøkrid K, Sletner L (2012). Impact of ethnicity on gestational diabetes identified with the WHO and the modified International Association of Diabetes and Pregnancy Study Groups criteria: a population-based cohort study. Eur J Endocrinol.

[24] McKeigue PM, Pierpoint T, Ferrie JE, Marmot MG (1992). Relationship of glucose intolerance and hyperinsulinaemia to body fat pattern in South Asians and Europeans. Diabetologia.

[25] Scarborough P, Bhatnagar P, Kaur A, Smolina K, Wickramasinghe K, Rayner M. Ethnic differences in cardiovascular disease 2010 edition. 2010.

[26] West J, Lawlor DA, Fairley L, Bhopal R, Cameron N, McKinney PA, Sattar N, Wright J (2013). UK-born Pakistani-origin infants are relatively more adipose than white British infants: findings from 8704 mother-offspring pairs in the born-in-bradford prospective birth cohort. J Epidemiol Community Health.

[27] Lawlor DA, West J, Fairley L, Nelson SM, Bhopal RS, Tuffnell D, Freeman DJ, Wright J, Whitelaw DC, Sattar N (2014). Pregnancy glycaemia and cord-blood levels of insulin and leptin in Pakistani and white British mother-offspring pairs: findings from a prospective pregnancy cohort. Diabetologia.

[28] Department for Communities and Local Government. The english indices of deprivation 2015 statistical release. 2015. https://www.gov.uk/government/statistics/english-indices-of-deprivation-2015. Accessed 1 Jan 2019.

[29] Wright J, Small N, Raynor P (2013). Cohort profile: The born in bradford multi-ethnic family cohort study. Int J Epidemiol.

[30] National Health Service. The General Practice Physical Activity Questionnaire (GPPAQ) A screening tool to assess adult physical activity levels, within primary care. 2009. https://www.gov.uk/government/uploads/system/uploads/attachment_data/file/192453/GPPAQ_-_guidance.pdf. Accessed 12 Dec 2017.

[31] Ahmad S, Harris T, Limb E (2015). Evaluation of reliability and validity of the General Practice Physical Activity Questionnaire (GPPAQ) in 60–74 year old primary care patients. BMC Fam Pract.

[32] Bull F, Milton K, Boehler C. Evaluation of the physical activity care pathway London feasibility pilot—final technical report. 2008.

[33] World Health Organization. Definition, diagnosis and classification of diabetes mellitus. Part 1: diagnosis and classification of diabetes mellitus. 1999.

[34] National Collaborating Centre for Women’s and Children’s Health. Antenatal care: routine care for the healthy pregnant woman. 2008. https://www.ncbi.nlm.nih.gov/books/NBK51886/pdf/Bookshelf_NBK51886.pdf. Accessed 1 Jan 2019.

[35] Wallace AM, McMahon AD, Packard CJ, Kelly A, Shepherd J, Gaw A, Sattar N (2001). Plasma leptin and the risk of cardiovascular disease in the west of Scotland coronary prevention study (WOSCOPS). Circulation.

[36] West J, Manchester B, Wright J, Lawlor DA, Waiblinger D (2011). Reliability of routine clinical measurements of neonatal circumferences and research measurements of neonatal skinfold thicknesses: findings from the Born in Bradford study. Paediatr Perinat Epidemiol.

[37] Fairley L, Cabieses B, Small N, Petherick ES, Lawlor DA, Pickett KE, Wright J (2014). Using latent class analysis to develop a model of the relationship between socioeconomic position and ethnicity: cross-sectional analyses from a multi-ethnic birth cohort study. BMC Public Health.

[38] Goldberg DP, Hillier VF (1979). A scaled version of the General Health Questionnaire. Psychol Med.

[39] Peacock A, Hutchinson D, Wilson J, McCormack C, Bruno R, Olsson CA, Allsop S, Elliott E, Burns L, Mattick RP (2018). Adherence to the caffeine intake guideline during pregnancy and birth outcomes: a prospective cohort study. Nutrients.

[40] White IR, Royston P, Wood AM (2011). Multiple imputation using chained equations: issues and guidance for practice. Stat Med.

[41] Kontopantelis E, White IR, Sperrin M, Buchan I (2017). Outcome-sensitive multiple imputation: a simulation study. BMC Med Res Methodol.

[42] Petherick ES, Tuffnell D, Wright J (2015). Experiences and outcomes of maternal Ramadan fasting during pregnancy: results from a sub-cohort of the Born in Bradford birth cohort study. BMC Pregnancy Childbirth.

[43] West J, Lawlor DA, Fairley L, Wright J (2014). Differences in socioeconomic position, lifestyle and health-related pregnancy characteristics between Pakistani and White British women in the Born in Bradford prospective cohort study: the influence of the woman’s, her partner’s and their parents’ place. BMJ Open.

[44] Aune D, Sen A, Henriksen T, Saugstad OD, Tonstad S (2016). Physical activity and the risk of gestational diabetes mellitus: a systematic review and dose–response meta-analysis of epidemiological studies. Eur J Epidemiol.

[45] Gill JMR, Celis-Morales CA, Ghouri N (2014). Physical activity, ethnicity and cardio-metabolic health: does one size fit all?. Atherosclerosis.

[46] Butler CL, Williams MA, Sorensen TK, Frederick IO, Leisenring WM (2004). Relation between maternal recreational physical activity and plasma lipids in early pregnancy. Am J Epidemiol.

[47] Loprinzi PD, Fitzgerald EM, Woekel E, Cardinal BJ (2013). Association of physical activity and sedentary behavior with biological markers among U.S. pregnant women. J Women’s Heal.

[48] Hesketh KR, Evenson KR, Stroo M, Clancy SM, Østbye T, Benjamin-Neelon SE (2018). Physical activity and sedentary behavior during pregnancy and postpartum, measured using hip and wrist-worn accelerometers. Prev Med Reports.

[49] Mourtakos SP, Tambalis KD, Panagiotakos DB, Antonogeorgos G, Arnaoutis G, Karteroliotis K, Sidossis LS (2015). Maternal lifestyle characteristics during pregnancy, and the risk of obesity in the offspring: a study of 5,125 children. BMC Pregnancy Childbirth.

[50] Clapp J, Kiess W (1998). Cord blood leptin reflects fetal fat mass. J Soc Gynecol Investig.

[51] Bisson M, Tremblay F, St-Onge O, Robitaille J, Pronovost E, Simonyan D, Marc I (2017). Influence of maternal physical activity on infant’s body composition. Pediatr Obes.

[52] Dahly DL, Li X, Smith HA, Khashan AS, Murray DM, Kiely ME, O’Bhourihane J, McCarthy FP, Kenny LC, Kearney PM (2018). Associations between maternal lifestyle factors and neonatal body composition in the Screening for Pregnancy Endpoints (Cork) cohort study. Int J Epidemiol.

[53] Juhl M, Olsen J, Andersen PK, Nøhr EA, Andersen A-MN (2010). Physical exercise during pregnancy and fetal growth measures: a study within the Danish National Birth Cohort. Am J Obstet Gynecol.

[54] Lindqvist M, Lindkvist M, Eurenius E, Persson M, Ivarsson A, Mogren I (2016). Leisure time physical activity among pregnant women and its associations with maternal characteristics and pregnancy outcomes. Sex Reprod Healthc.

[55] Watson ED, Brage S, White T, Westgate K, Norris SA, Van Poppel MNM, Micklesfield LK (2018). The influence of objectively measured physical activity during pregnancy on maternal and birth outcomes in urban black south african women. Matern Child Health J.

