# Supplementary material for: Associations of Pregnancy Physical Activity with Maternal Cardiometabolic Health, Neonatal Delivery Outcomes and Body Composition in a Biethnic Cohort of 7305 Mother–Child Pairs: The Born in Bradford Study
Source: Sports Med. 2019 Sep 26;50(3):615–28. doi: 10.1007/s40279-019-01193-8 (PMC7018786; doi:10.1007/s40279-019-01193-8)
Supplement: Supplementary file 1 — Supplementary material 1 (DOCX 89 kb) [file 40279_2019_1193_MOESM1_ESM.docx]

**Title:** Associations of pregnancy physical activity with maternal cardiometabolic health, neonatal delivery outcomes and body composition in a biethnic cohort of 7,305 mother-child pairs: the Born in Bradford study

**Authors:** Paul J Collings^1,2^, Diane Farrar^1^, Joanna Gibson^1^, Jane West^1^, Sally E. Barber^1^, John Wright^1^

**Affiliations:** ^1^Bradford Institute for Health Research, UK; ^2^Department of Health Sciences, University of York, UK

**Contact:** [paul.collings@bthft.nhs.uk](mailto:paul.collings@bthft.nhs.uk)

**Reference:** National Health Service. The General Practice Physical Activity Questionnaire

(GPPAQ) A screening tool to assess adult physical activity levels, within primary care.

2009. Available from: https://www.gov.uk/government/uploads/system/uploads/attachment_data/file/192453

/GPPAQ_-_guidance.pdf

**Calculating the four-level physical activity index:**

**Inactive**: Sedentary job and no physical exercise or cycling

**Somewhat active:** Sedentary job and some but <1 hour physical exercise and / or cycling per week OR Standing job and no physical exercise or cycling

**Moderately active:** Sedentary job and 1-2.9 hours physical exercise and / or cycling per week OR Standing job and some but <1 hour physical exercise and / or cycling per week OR Physical job and no physical exercise or cycling

**Active:** Sedentary job and ≥3 hours physical exercise and / or cycling per week OR Standing job and 1-2.9 hours physical exercise and / or cycling per week OR Physical job and some but <1 hour physical exercise and / or cycling per week OR Heavy manual job OR ≥3 hours brisk or fast pace walking per week
