# Supplementary material for: Associations of Pregnancy Physical Activity with Maternal Cardiometabolic Health, Neonatal Delivery Outcomes and Body Composition in a Biethnic Cohort of 7305 Mother–Child Pairs: The Born in Bradford Study
Source: Sports Med. 2019 Sep 26;50(3):615–28. doi: 10.1007/s40279-019-01193-8 (PMC7018786; doi:10.1007/s40279-019-01193-8)
Supplement: Supplementary file 2 — Supplementary material 2 (DOCX 33 kb) [file 40279_2019_1193_MOESM2_ESM.docx]

**Supplementary file 2 – Full details of results shown in the figures and complete case results**

**Title:** Associations of pregnancy physical activity with maternal cardiometabolic health, neonatal delivery outcomes and body composition in a biethnic cohort of 7,305 mother-child pairs: the Born in Bradford study

**Authors:** Paul J Collings^1,2^, Diane Farrar^1^, Joanna Gibson^1^, Jane West^1^, Sally E. Barber^1^, John Wright^1^

**Affiliations:** ^1^Bradford Institute for Health Research, UK; ^2^Department of Health Sciences, University of York, UK

**Contact:** paul.collings@bthft.nhs.uk

**Table 1. Full details of results for associations of mid-pregnancy physical activity with maternal adiposity (illustrated in Figure 1) and late-pregnancy gestational weight gain (illustrated in Figure 2)**

|  |  | Participants: observations  (*n* in each activity category) | Inactive | Somewhat active | Moderately active | Active | *p*-trend |
| --- | --- | --- | --- | --- | --- | --- | --- |
| White British | Triceps skinfold (mm) | 1662: 1700  (694 / 405 / 346 / 255) | 26.5 (25.9 to 27.0)  Ref | 25.9 (25.2 to 26.6)  0.20 | 26.5 (25.9 to 27.0)  0.38 | **25.1 (24.2 to 25.9**)  **0.007** | **0.016** |
|  | Mid-upper arm circumference (cm) | 1673: 1712  (700 / 409 / 348 / 255) | 30.3 (30.0 to 30.7)  Ref | 30.6 (30.2 to 31.1)  0.32 | 30.6 (30.1 to 31.1)  0.39 | **29.5 (29.1 to 30.0**)  **0.012** | 0.11 |
|  | Late-pregnancy gestational weight gain (kg) | 1120: 1137  (424 / 286 / 260 / 167) | 5.5 (5.2 to 5.7)  Ref | 5.4 (5.1 to 5.6)  0.60 | 5.3 (5.0 to 5.5)  0.29 | 5.4 (5.1 to 5.7)  0.68 | 0.45 |
| Pakistani-origin | Triceps skinfold (mm) | 585: 590  (424 / 88 / 41 / 37) | 24.6 (23.9 to 25.3)  Ref | 24.2 (22.7 to 25.7)  0.64 | 25.4 (23.0 to 27.8)  0.53 | 24.1 (21.7 to 26.5)  0.72 | 0.95 |
|  | Mid-upper arm circumference (cm) | 590: 595  (427 / 88 / 42 / 38) | 28.3 (27.9 to 28.6)  Ref | 28.5 (27.7 to 29.3)  0.67 | **29.8 (28.5 to 31.1)**  **0.032** | 29.0 (27.7 to 30.3)  0.31 | 0.067 |
|  | Late-pregnancy gestational weight gain (kg) | 1582: 1628  (1181 / 245 / 92 / 110) | 4.9 (4.8 to 5.0)  Ref | 4.9 (4.7 to 5.2)  0.72 | 4.8 (4.5 to 5.2)  0.93 | 5.0 (4.6 to 5.4)  0.59 | 0.63 |

Data are estimated marginal means (95% confidence interval) adjusted for maternal age, gestational age at measurement, socioeconomic status, parity, maternal smoking, alcohol consumption, caffeine intake, sleep quality, and use of dietary supplements. The results for late-pregnancy gestational weight gain are further adjusted for maternal early-pregnancy BMI and the number of weeks between mid- and late- pregnancy weight measurements. Below the estimates are *p*-values. Bold font denotes significantly different values compared to the referent inactive group (*p*<0.05) or across physical activity categories (*p*-trend<0.05).

**Table 2. Complete-case associations of mid-pregnancy physical activity with maternal adiposity and late-pregnancy gestational weight gain**

|  |  | Participants: observations  (*n* in each activity category) | Inactive | Somewhat active | Moderately active | Active | *p*-trend |
| --- | --- | --- | --- | --- | --- | --- | --- |
| White British | Triceps skinfold (mm) | 1604: 1639  (661 / 393 / 339 / 246) | 26.5 (25.9 to 27.0)  Ref | 25.9 (25.2 to 26.6)  0.24 | 26.1 (25.3 to 26.8)  0.42 | **25.2 (24.4 to 26.0**)  **0.015** | **0.030** |
|  | Mid-upper arm circumference (cm) | 1614: 1650  (667 / 397 / 340 / 246) | 30.3 (30.0 to 30.7)  Ref | 30.6 (30.2 to 31.1)  0.29 | 30.5 (30.0 to 31.0)  0.55 | **29.6 (29.1 to 30.1**)  **0.016** | 0.092 |
|  | Late-pregnancy gestational weight gain (kg) | 1081: 1097  (405 / 281 / 249 / 162) | 5.5 (5.2 to 5.7)  Ref | 5.4 (5.1 to 5.6)  0.69 | 5.3 (5.0 to 5.5)  0.26 | 5.3 (5.0 to 5.6)  0.50 | 0.32 |
| Pakistani-origin | Triceps skinfold (mm) | 507: 512  (350 / 87 / 39 / 36) | 24.8 (24.0 to 25.5)  Ref | 24.3 (22.8 to 25.8)  0.58 | 25.4 (23.1 to 27.7)  0.59 | 23.7 (21.4 to 26.1)  0.42 | 0.65 |
|  | Mid-upper arm circumference (cm) | 512: 517  (353 / 87 / 40 / 37) | 28.2 (27.8 to 28.6)  Ref | 28.4 (27.6 to 29.2)  0.66 | **29.7 (28.4 to 31.0)**  **0.041** | 28.8 (27.5 to 30.2)  0.40 | 0.10 |
|  | Late-pregnancy gestational weight gain (kg) | 1436: 1475  (1048 / 234 / 88 / 105) | 4.9 (4.8 to 5.0)  Ref | 4.9 (4.7 to 5.2)  0.89 | 4.9 (4.5 to 5.3)  0.88 | 5.0 (4.6 to 5.4)  0.60 | 0.70 |

Data are estimated marginal means (95% confidence interval) adjusted for maternal age, gestational age at measurement, socioeconomic status, parity, maternal smoking, alcohol consumption, caffeine intake, sleep quality, and use of dietary supplements. The results for late-pregnancy gestational weight gain are further adjusted for maternal early-pregnancy BMI and the number of weeks between mid- and late- pregnancy weight measurements. Below the estimates are *p*-values. Bold font denotes significantly different values compared to the referent inactive group (*p*<0.05) or across physical activity categories (*p*-trend<0.05).

**Table 3. Complete-case associations of mid-pregnancy physical activity with maternal insulin and glucose, and blood pressure**

|  |  | Participants: observations  (*n* in each activity category) | Inactive | Somewhat active | Moderately active | Active | *p*-trend |
| --- | --- | --- | --- | --- | --- | --- | --- |
| White British | Fasting insulin (pmol/l) | 2915: 3017  (1156 / 730 / 656 / 475) | 72.4 (70.6 to 74.2)  Ref | 72.4 (70.1 to 74.7)  0.99 | 70.2 (68.0 to 72.5)  0.14 | **68.2 (65.8 to 70.7)**  **0.008** | **0.006** |
|  | Fasting glucose (mmol/l) | 2954: 3059  (1181 / 742 / 653 / 483) | 4.4 (4.3 to 4.4)  Ref | 4.4 (4.3 to 4.4)  0.45 | 4.4 (4.3 to 4.4)  0.36 | 4.4 (4.3 to 4.4)  0.85 | 0.74 |
|  | Postload glucose (mmol/l) | 2952: 3057  (1180 / 742 / 652 / 483) | 5.4 (5.3 to 5.4)  Ref | 5.3 (5.2 to 5.4)  0.20 | 5.3 (5.2 to 5.4)  0.12 | **5.2 (5.1 to 5.3)**  **0.012** | **0.010** |
|  | Systole (mmHg) | 2982: 3085  (1182 / 746 / 668 / 489) | 113 (112 to 113)  Ref | 112 (111 to 113)  0.41 | 112 (112 to 113)  0.82 | 112 (111 to 113)  0.51 | 0.59 |
|  | Diastole (mmHg) | 2982: 3086  (1183 / 746 / 668 / 489) | 66 (66 to 67)  Ref | 66 (65 to 66)  0.29 | 66 (65 to 67)  0.39 | 66 (65 to 67)  0.88 | 0.70 |
| Pakistani-origin | Fasting insulin (pmol/l) | 2934: 3077  (2210 / 457 / 208 / 202) | 87.4 (85.7 to 89.1)  Ref | 86.4 (82.7 to 89.1)  0.66 | 84.2 (79.5 to 89.2)  0.23 | 84.2 (80.9 to 91.9)  0.70 | 0.37 |
|  | Fasting glucose (mmol/l) | 3155: 3324  (2385 / 499 / 224 / 216) | 4.6 (4.5 to 4.6)  Ref | 4.6 (4.5 to 4.6)  0.80 | 4.6 (4.5 to 4.6)  0.56 | 4.6 (4.5 to 4.6)  0.76 | 0.64 |
|  | Postload glucose (mmol/l) | 3154: 3322  (2384 / 498 / 224 / 216) | 5.7 (5.7 to 5.8)  Ref | 5.6 (5.5 to 5.7)  0.12 | 5.5 (5.4 to 5.7)  0.11 | 5.6 (5.4 to 5.8)  0.40 | 0.094 |
|  | Systole (mmHg) | 3137: 3296  (2363 / 495 / 221 / 217) | 107 (107 to 108)  Ref | **106 (105 to 107)**  **0.029** | 107 (105 to 108)  0.55 | 108 (107 to 109)  0.36 | 0.99 |
|  | Diastole (mmHg) | 3137: 3296  (2363 / 495 / 221 / 217) | 64 (63 to 64)  Ref | 63 (63 to 64)  0.12 | 64 (63 to 65)  0.63 | 64 (63 to 65)  0.84 | 0.71 |

Data are estimated marginal means (95% confidence interval) adjusted for maternal age, gestational age at measurement, socioeconomic status, parity, maternal smoking, alcohol consumption, caffeine intake, sleep quality, use of dietary supplements, and maternal early-pregnancy BMI. Below the estimates are *p*-values. Bold font denotes significantly different values compared to the referent inactive group (*p*<0.05) or across physical activity categories (*p*-trend<0.05).

**Table 4. Complete-case associations of mid-pregnancy physical activity with maternal lipid and lipoprotein cholesterol**

|  |  | Participants: observations  (*n* in each activity category) | Inactive | Somewhat active | Moderately active | Active | *p*-trend |
| --- | --- | --- | --- | --- | --- | --- | --- |
| White British | Total cholesterol  (mmol/l) | 2914: 3016  (1155 / 730 / 656 / 475) | 6.4 (6.4 to 6.5)  Ref | 6.4 (6.4 to 6.5)  0.70 | 6.4 (6.3 to 6.5)  0.57 | 6.4 (6.3 to 6.5)  0.47 | 0.38 |
|  | Triglycerides  (mmol/l) | 2914: 3016  (1155 / 730 / 656 / 475) | 1.9 (1.9 to 2.0)  Ref | 1.9 (1.8 to 1.9)  0.30 | **1.8 (1.8 to 1.9)**  **0.006** | **1.8 (1.8 to 1.9)**  **0.005** | **0.001** |
|  | LDL cholesterol  (mmol/l) | 2893: 2994  (1144 / 725 / 652 / 473) | 3.5 (3.5 to 3.6)  Ref | 3.6 (3.5 to 3.7)  0.30 | 3.6 (3.5 to 3.6)  0.75 | 3.5 (3.4 to 3.6)  0.85 | 0.91 |
|  | HDL cholesterol  (mmol/l) | 2914: 3016  (1155 / 730 / 656 / 475) | 2.0 (1.9 to 2.0)  Ref | 2.0 (1.9 to 2.0)  0.65 | 2.0 (1.9 to 2.0)  0.75 | 2.0 (2.0 to 2.1)  0.44 | 0.60 |
|  | Total : HDL ratio | 2914: 3016  (1155 / 730 / 656 / 475) | 3.3 (3.2 to 3.4)  Ref | 3.3 (3.2 to 3.4)  0.80 | 3.3 (3.2 to 3.4)  0.75 | 3.2 (3.2 to 3.3)  0.16 | 0.20 |
| Pakistani-origin | Total cholesterol  (mmol/l) | 2936: 3079  (2212 / 457 / 208 / 202) | 6.0 (6.0 to 6.1)  Ref | 6.0 (5.9 to 6.1)  0.78 | 6.0 (5.9 to 6.1)  0.62 | 6.1 (5.9 to 6.2)  0.76 | 0.97 |
|  | Triglycerides  (mmol/l) | 2936: 3079  (2212 / 457 / 208 / 202) | 1.9 (1.8 to 1.9)  Ref | 1.8 (1.8 to 1.9)  0.15 | 1.8 (1.7 to 1.9)  0.24 | 1.9 (1.8 to 1.9)  0.74 | 0.28 |
|  | LDL cholesterol  (mmol/l) | 2918: 3060  (2200 / 453 / 207 / 200) | 3.2 (3.1 to 3.2)  Ref | 3.2 (3.1 to 3.3)  0.65 | 3.2 (3.0 to 3.3)  0.56 | 3.2 (3.1 to 3.4)  0.71 | 0.94 |
|  | HDL cholesterol  (mmol/l) | 2936: 3079  (2212 / 457 / 208 / 202) | 1.9 (1.9 to 2.0)  Ref | 2.0 (1.9 to 2.1)  0.094 | 2.0 (1.9 to 2.1)  0.48 | 2.0 (1.9 to 2.1)  0.5 | 0.29 |
|  | Total : HDL ratio | 2936: 3079  (2212 / 457 / 208 / 202) | 3.2 (3.1 to 3.2)  Ref | 3.1 (3.0 to 3.2)  0.084 | 3.1 (3.0 to 3.2)  0.16 | 3.2 (3.1 to 3.3)  0.94 | 0.31 |

Data are estimated marginal means (95% confidence interval) adjusted for maternal age, gestational age at measurement, socioeconomic status, parity, maternal smoking, alcohol consumption, caffeine intake, sleep quality, use of dietary supplements, and maternal early-pregnancy BMI. Below the estimates are *p*-values. Bold font denotes significantly different values compared to the referent inactive group (*p*<0.05) or across physical activity categories (*p*-trend<0.05).

**Table 5. Complete-case associations of mid-pregnancy physical activity with gestational age at birth, birth weight and offspring adiposity**

|  |  | Participants: observations  (*n* in each activity category) | Inactive | Somewhat active | Moderately active | Active | *p*-trend |
| --- | --- | --- | --- | --- | --- | --- | --- |
| White British | Gestational age (months) | 3063: 3172  (1226 / 763 / 683 / 500) | 39.6 (39.5 to 39.7)  Ref | 39.7 (39.5 to 39.8)  0.12 | 39.6 (39.4 to 39.7)  0.90 | 39.7 (39.5 to 39.8)  0.35 | 0.53 |
|  | Birth weight (g) | 3062: 3171  (1226 / 763 / 682 / 500) | 3373 (3349 to 3397)  Ref | 3342 (3312 to 3371)  0.12 | 3368 (3336 to 3400)  0.82 | 3357 (3320 to 3394)  0.49 | 0.62 |
|  | Sum of skinfolds (mm) | 2141: 2187  (866 / 537 / 445 / 339) | 10.1 (10.0 to 10.3)  Ref | 10.1 (9.9 to 10.2)  0.42 | **9.9 (9.7 to 10.1)**  **0.018** | 9.9 (9.7 to 10.1)  0.081 | **0.018** |
|  | Triceps skinfold (mm) | 2150: 2196  (871 / 538 / 447 / 340) | 5.2 (5.2 to 5.3)  Ref | 5.2 (5.1 to 5.3)  0.88 | 5.1 (5.0 to 5.2)  0.11 | 5.2 (5.0 to 5.3)  0.19 | 0.082 |
|  | Subscapular skinfold (mm) | 2142: 2188  (867 / 537 / 445 / 339) | 4.9 (4.8 to 5.0)  Ref | 4.8 (4.7 to 4.9)  0.16 | **4.7 (4.6 to 4.8)**  **0.004** | 4.8 (4.7 to 4.9)  0.057 | **0.008** |
|  | Abdominal circumference (cm) | 2712: 2779  (1086 / 675 / 582 / 436) | 32.0 (31.8 to 32.1)  Ref | 31.9 (31.8 to 32.1)  0.66 | 32.1 (31.9 to 32.3)  0.23 | 31.9 (31.7 to 32.1)  0.62 | 0.85 |
|  | Mid-upper arm circumference (cm) | 2714: 2781  (1088 / 673 / 582 / 438) | 10.9 (10.8 to 10.9)  Ref | 10.8 (10.7 to 10.9)  0.13 | 10.8 (10.7 to 10.9)  0.21 | 10.8 (10.7 to 10.9)  0.078 | 0.066 |
|  | Cord leptin (ng/ml) | 1839: 1880  (753 / 440 / 407 / 280) | 6.6 (6.2 to 6.9)  Ref | 6.6 (6.1 to 7.1)  0.97 | 6.8 (6.2 to 7.3)  0.55 | 6.7 (6.1 to 7.4)  0.61 | 0.50 |
|  | Cord insulin (pmol/l) | 1833: 1873  (752 / 438 / 405 / 278) | 3.6 (3.4 to 3.8)  Ref | 3.6 (3.3 to 3.9)  0.98 | 3.5 (3.2 to 3.8)  0.51 | 3.5 (3.1 to 3.8)  0.54 | 0.43 |
| Pakistani-origin | Gestational age (months) | 3252: 3428  (2462 / 513 / 231 / 222) | 39.4 (39.3 to 39.5)  Ref | 39.4 (39.2 to 39.6)  0.77 | 39.4 (39.1 to 39.7)  0.95 | 39.5 (39.3 to 39.7)  0.52 | 0.70 |
|  | Birth weight (g) | 3252: 3428  (2462 / 513 / 231 / 222) | 3143 (3127 to 3159)  Ref | 3108 (3073 to 3143)  0.076 | 3161 (3102 to 3220)  0.56 | 3141 (3090 to 3191)  0.94 | 0.89 |
|  | Sum of skinfolds (mm) | 2521: 2601  (1889 / 377 / 166 / 169) | 9.6 (9.5 to 9.7)  Ref | 9.6 (9.4 to 9.7)  0.49 | 9.8 (9.5 to 10.1)  0.42 | 9.7 (9.5 to 10.0)  0.49 | 0.46 |
|  | Triceps skinfold (mm) | 2530: 2610  (1896 / 378 / 167 / 169) | 5.0 (5.0 to 5.1)  Ref | 5.0 (4.9 to 5.1)  0.42 | 5.1 (4.9 to 5.2)  0.59 | 5.0 (4.9 to 5.2)  0.92 | 0.89 |
|  | Subscapular skinfold (mm) | 2523: 2603  (1890 / 378 / 166 / 169) | 4.6 (4.6 to 4.7)  Ref | 4.6 (4.5 to 4.7)  0.64 | 4.7 (4.5 to 4.9)  0.32 | 4.7 (4.5 to 4.9)  0.27 | 0.22 |
|  | Abdominal circumference (cm) | 2887: 3011  (2175 / 448 / 198 / 190) | 30.7 (30.6 to 30.8)  Ref | 30.4 (30.2 to 30.7)  0.057 | 30.3 (29.9 to 30.7)  0.092 | 30.8 (30.4 to 31.1)  0.68 | 0.39 |
|  | Mid-upper arm circumference (cm) | 2882: 3005  (2170 / 448 / 197 / 190) | 10.5 (10.5 to 10.6)  Ref | **10.4 (10.3 to 10.5)**  **0.028** | 10.5 (10.4 to 10.7)  0.69 | 10.5 (10.4 to 10.7)  0.83 | 0.42 |
|  | Cord leptin (ng/ml) | 1970: 2030  (1467 / 293 / 143 / 127) | 7.5 (7.2 to 7.8)  Ref | **6.4 (5.8 to 7.1)**  **0.004** | 6.9 (6.0 to 8.0)  0.28 | 7.4 (6.3 to 8.7)  0.88 | 0.23 |
|  | Cord insulin (pmol/l) | 1954: 2013  (1455 / 292 / 142 / 124) | 4.1 (3.9 to 4.2)  Ref | 3.9 (3.6 to 4.3)  0.51 | 3.9 (3.5 to 4.5)  0.66 | 3.8 (3.3 to 4.4)  0.43 | 0.34 |

Data are estimated marginal means (95% confidence interval) adjusted for maternal age, gestational age at measurement, socioeconomic status, parity, gestational age (when itself was not an outcome), child sex, mode of delivery, maternal smoking, alcohol consumption, caffeine intake, sleep quality, use of dietary supplements, and maternal early-pregnancy BMI. Below the estimates are *p*-values. Bold font denotes significantly different values compared to the referent inactive group (*p*<0.05) or across physical activity categories (*p*-trend<0.05).
